# Dibromido(2,9-dimethyl-1,10-phenanthroline-κ^2^ *N*,*N*′)mercury(II)

**DOI:** 10.1107/S1600536809009994

**Published:** 2009-04-02

**Authors:** Robabeh Alizadeh, Amene Heidari, Roya Ahmadi, Vahid Amani

**Affiliations:** a School of Chemistry, Damghan University of Basic Sciences, Damghan, Iran; b Department of Chemistry, University of Zabol, Zabol, Iran; c Islamic Azad University, Shahr-e-Rey Branch, Tehran, Iran

## Abstract

In the mol­ecule of the title compound, [HgBr_2_(C_14_H_12_N_2_)], the Hg^II^ atom is four-coordinated in a distorted tetra­hedral configuration by two N atoms from a 2,9-dimethyl-1,10-phenanthroline ligand and by two Br atoms. In the crystal structure, weak inter­molecular C—H⋯Br hydrogen bonds link the mol­ecules into chains along the *b* axis. There are π–π contacts between the phenanthroline rings [centroid–centroid distances = 3.806 (4), 3.819 (4), 3.739 (3), 3.690 (3), 3.619 (4) and 3.674 (3) Å].

## Related literature

For related structures, see: Ahmadi *et al.* (2008 ▶); Craig *et al.* (1974 ▶); Hughes *et al.* (1985 ▶); Kalateh, Ebadi *et al.* (2008 ▶); Kalateh, Norouzi *et al.* (2008 ▶); Perlepes *et al.* (1995 ▶); Tadayon Pour *et al.* (2008 ▶); Xie *et al.* (2004 ▶); Yousefi *et al.* (2009 ▶); Yousefi, Rashidi Vahid *et al.* (2008 ▶); Yousefi, Tadayon Pour *et al.* (2008 ▶). For bond-length data, see: Allen *et al.* (1987 ▶).
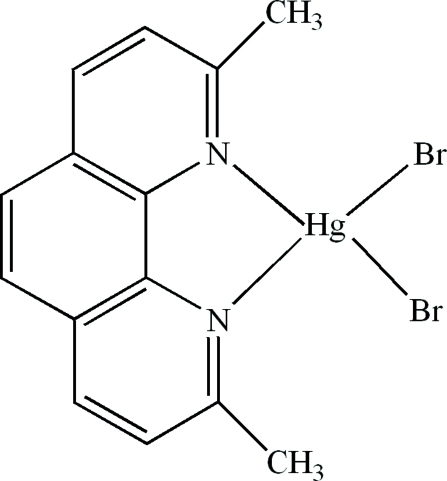


## Experimental

### 

#### Crystal data

[HgBr_2_(C_14_H_12_N_2_)]
*M* *_r_* = 568.65
Monoclinic, 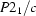

*a* = 7.8587 (7) Å
*b* = 10.5556 (8) Å
*c* = 18.7304 (13) Å
β = 97.517 (6)°
*V* = 1540.4 (2) Å^3^
*Z* = 4
Mo *K*α radiation
μ = 15.17 mm^−1^
*T* = 298 K
0.49 × 0.44 × 0.26 mm

#### Data collection

Bruker SMART CCD area-detector diffractometer
Absorption correction: multi-scan (*SADABS*; Sheldrick, 1998 ▶)*T*_min_ = 0.008, *T*_max_ = 0.022
11121 measured reflections
4161 independent reflections
3006 reflections with *I* > 2σ(*I*)
*R*_int_ = 0.093

#### Refinement

*R*[*F* ^2^ > 2σ(*F* ^2^)] = 0.068
*wR*(*F* ^2^) = 0.183
*S* = 1.12
4161 reflections
172 parameters
H-atom parameters constrained
Δρ_max_ = 1.23 e Å^−3^
Δρ_min_ = −2.40 e Å^−3^

### 

Data collection: *SMART* (Bruker, 1998 ▶); cell refinement: *SAINT* (Bruker, 1998 ▶); data reduction: *SAINT*; program(s) used to solve structure: *SHELXTL* (Sheldrick, 2008 ▶); program(s) used to refine structure: *SHELXTL*; molecular graphics: *ORTEP-3 for Windows* (Farrugia, 1997 ▶); software used to prepare material for publication: *WinGX* (Farrugia, 1999 ▶).

## Supplementary Material

Crystal structure: contains datablocks I, global. DOI: 10.1107/S1600536809009994/hk2645sup1.cif

Structure factors: contains datablocks I. DOI: 10.1107/S1600536809009994/hk2645Isup2.hkl

Additional supplementary materials:  crystallographic information; 3D view; checkCIF report

## Figures and Tables

**Table:** 

Hg1—Br2	2.5053 (16)
Hg1—Br1	2.5156 (17)
N1—Hg1	2.345 (8)
N2—Hg1	2.340 (8)

**Table:** 

Br2—Hg1—Br1	116.23 (6)
N1—Hg1—Br1	109.6 (2)
N1—Hg1—Br2	115.7 (2)
N2—Hg1—Br1	117.3 (2)
N2—Hg1—Br2	118.2 (2)
N2—Hg1—N1	71.2 (3)

**Table 2:** Hydrogen-bond geometry (Å, °)

*D*—H⋯*A*	*D*—H	H⋯*A*	*D*⋯*A*	*D*—H⋯*A*
C1—H1*C*⋯Br2^i^	0.96	2.85	3.812 (18)	178

Symmetry code: (i) 
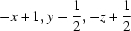
.

**Fig. 1.**
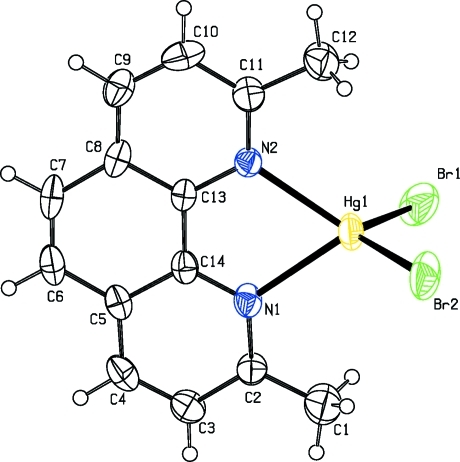
The molecular structure of the title molecule, with the atom-numbering scheme. Displacement ellipsoids are drawn at the 40% probability level.

**Fig. 2.**
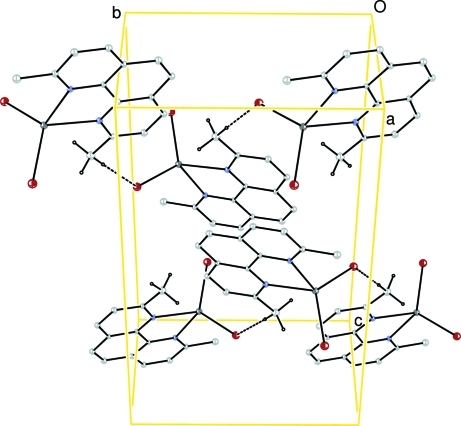
A partial packing diagram of the title compound. Hydrogen bonds are shown as dashed lines. H atoms not involved in hydrogen bonding have been omitted.

## supplementary crystallographic information

### Comment

There are several Hg^II^ complexes, with formula, [Hg(N—N)*X*_2_], (*X*=Br, Cl and I), such as [Hg(TPA)Br_2_], (II), (Xie *et al.*, 2004), [Hg(TPD)Br_2_], (III), (Hughes *et al.*, 1985), [Hg(NH(py)_2_)Br_2_], (IV), (Kalateh, Norouzi *et al.*, 2008), [Hg(6-mbpy)Cl_2_], (V), (Ahmadi *et al.*, 2008), [Hg(NH(py)_2_)Cl_2_], (VI), (Yousefi, Allahgholi Ghasri *et al.*, 2009), [Hg(4,4'-dmbpy)I_2_], (VII), (Yousefi, Tadayon Pour *et al.*, 2008), [Hg(5,5'-dmbpy)I_2_], (VIII), (Tadayon Pour *et al.*, 2008) and [Hg(dmphen)I_2_], (IX), (Yousefi, Rashidi Vahid *et al.*, 2008) [where TPA is tris(2-pyridyl)amine, TPD is *N*,*N*,*N*',*N*'-Tetramethyl-*o*-phenylenediamine, NH(py)_2_ is di-2-pyridylamine, 6-mbpy is 6-methyl-2,2'-bipyridine, 4,4'-dmbpy is 4,4'-dimethyl-2,2'-bipyridine, 5,5'-dmbpy is 5,5'-dimethyl-2,2'-bipyridine and dmphen is 4,7-diphenyl-1,10-phenanthroline] have been synthesized and characterized by single-crystal X-ray diffraction methods.

There are also several Hg^II^ dimer complexes, with formula, [{HgBr(N—N)}_2_(µ-Br)_2_], such as [{HgBr(bipy)}_2_(µ-Br)_2_], (*X*), (Craig *et al.*, 1974),[{HgBr(pquin)}_2_(µ-Br)_2_], (XI), (Perlepes *et al.*, 1995) and [{HgBr(4,4'-dmbpy)}_2_(µ-Br)_2_], (XII), (Kalateh, Ebadi *et al.*, 2008) [where bipy is 2,2'-bipyridine and pquin is 2-(2'-pyridyl)quinoxaline] have been synthesized and characterized by single-crystal X-ray diffraction methods. We report herein the synthesis and crystal structure of the title compound, (I).

In the title compound, (Fig. 1), the Hg^II^ atom is four-coordinated in a distorted tetrahedral configuration by two N atoms from 2,9-dimethyl-1,10-phenanthroline and by two Br atoms. The Hg-Br and Hg-N bond lengths (Allen *et al.*, 1987) and angles (Table 1) are within normal ranges, and comparable with the corresponding values in (II) and (III).

In the crystal structure, weak intermolecular C-H···Br hydrogen bonds (Table 2) link the molecules into chains along the b-axis, in which they may be effective in the stabilization of the crystal structure (Fig. 2). The π-π contacts between the phenanthroline rings, Cg3···Cg2^i^, Cg3···Cg3^ii^, Cg4···Cg1^i^, Cg4···Cg2^i^, Cg4···Cg3^ii^ and Cg4···Cg4^i^ [symmetry codes: (i) 1 - x, -y, -z; (ii) 2 - x, -y, -z, where Cg1, Cg2, Cg3 and Cg4 are centroids of the rings A (Hg1/N1/N2/C13/C14), B (N1/C2-C5/C14), C (N2/C8-C11/C13) and D (C5-C8/C13/C14), respectively] may further stabilize the structure, with centroid-centroid distances of 3.806 (4), 3.819 (4), 3.739 (3), 3.690 (3), 3.619 (4) and 3.674 (3) Å, respectively.

### Experimental

For the preparation of the title compound, (I), a solution of 2,9-dimethyl-1,10-phenanthroline (0.25 g, 1.20 mmol) in methanol (10 ml) was added to a solution of HgBr_2_ (0.43 g, 1.20 mmol) in methanol (20 ml) at room temperature. Crystals suitable for X-ray analysis were obtained by methanol diffusion to a colorless solution in DMSO and isolated after one week (yield; 0.51 g, 74.7%).

### Refinement

H atoms were positioned geometrically, with C-H = 0.93 and 0.96 Å for aromatic and methyl H and constrained to ride on their parent atoms, with U_iso_(H) = 1.2U_eq_(C).

### Crystal data

**Table:**

[HgBr_2_(C_14_H_12_N_2_)]	*F*(000) = 1040
*M**_r_* = 568.65	*D*_x_ = 2.452 Mg m^−^^3^
Monoclinic, *P*2_1_/*c*	Mo *K*α radiation, λ = 0.71073 Å
Hall symbol: -P 2ybc	Cell parameters from 1005 reflections
*a* = 7.8587 (7) Å	θ = 2.2–29.2°
*b* = 10.5556 (8) Å	µ = 15.17 mm^−^^1^
*c* = 18.7304 (13) Å	*T* = 298 K
β = 97.517 (6)°	Block, colorless
*V* = 1540.4 (2) Å^3^	0.49 × 0.44 × 0.26 mm
*Z* = 4	

### Data collection

**Table:**

Bruker SMART CCD area-detector diffractometer	4161 independent reflections
Radiation source: fine-focus sealed tube	3006 reflections with *I* > 2σ(*I*)
graphite	*R*_int_ = 0.093
φ and ω scans	θ_max_ = 29.2°, θ_min_ = 2.2°
Absorption correction: numerical Shape of crystal determined optically	*h* = −10→10
*T*_min_ = 0.008, *T*_max_ = 0.022	*k* = −13→14
11121 measured reflections	*l* = −19→25

### Refinement

**Table:**

Refinement on *F*^2^	Primary atom site location: structure-invariant direct methods
Least-squares matrix: full	Secondary atom site location: difference Fourier map
*R*[*F*^2^ > 2σ(*F*^2^)] = 0.068	Hydrogen site location: inferred from neighbouring sites
*wR*(*F*^2^) = 0.183	H-atom parameters constrained
*S* = 1.12	*w* = 1/[σ^2^(*F*_o_^2^) + (0.0825*P*)^2^ + 6.5584*P*] where *P* = (*F*_o_^2^ + 2*F*_c_^2^)/3
4161 reflections	(Δ/σ)_max_ = 0.007
172 parameters	Δρ_max_ = 1.23 e Å^−^^3^
0 restraints	Δρ_min_ = −2.40 e Å^−^^3^

### Special details

**Table:**

Geometry. All e.s.d.'s (except the e.s.d. in the dihedral angle between two l.s. planes) are estimated using the full covariance matrix. The cell e.s.d.'s are taken into account individually in the estimation of e.s.d.'s in distances, angles and torsion angles; correlations between e.s.d.'s in cell parameters are only used when they are defined by crystal symmetry. An approximate (isotropic) treatment of cell e.s.d.'s is used for estimating e.s.d.'s involving l.s. planes.
Refinement. Refinement of *F*^2^ against ALL reflections. The weighted *R*-factor *wR* and goodness of fit *S* are based on *F*^2^, conventional *R*-factors *R* are based on *F*, with *F* set to zero for negative *F*^2^. The threshold expression of *F*^2^ > σ(*F*^2^) is used only for calculating *R*-factors(gt) *etc*. and is not relevant to the choice of reflections for refinement. *R*-factors based on *F*^2^ are statistically about twice as large as those based on *F*, and *R*- factors based on ALL data will be even larger.

### Fractional atomic coordinates and isotropic or equivalent isotropic displacement parameters (Å^2^)

**Table:**

	*x*	*y*	*z*	*U*_iso_*/*U*_eq_	
Hg1	0.21080 (7)	0.79014 (4)	0.39095 (3)	0.05349 (18)	
Br1	−0.0605 (2)	0.8266 (2)	0.30622 (9)	0.0836 (5)	
Br2	0.4351 (2)	0.95948 (12)	0.39430 (9)	0.0783 (5)	
N1	0.3074 (11)	0.5823 (8)	0.3777 (5)	0.0424 (18)	
N2	0.1876 (11)	0.6779 (8)	0.4970 (5)	0.0376 (17)	
C1	0.376 (3)	0.6301 (15)	0.2566 (8)	0.086 (5)	
H1A	0.4518	0.6985	0.2730	0.103*	
H1B	0.2641	0.6632	0.2400	0.103*	
H1C	0.4200	0.5866	0.2179	0.103*	
C2	0.3645 (15)	0.5411 (10)	0.3164 (5)	0.043 (2)	
C3	0.4185 (17)	0.4150 (11)	0.3120 (7)	0.056 (3)	
H3	0.4595	0.3857	0.2706	0.067*	
C4	0.4101 (14)	0.3354 (11)	0.3692 (7)	0.052 (3)	
H4	0.4445	0.2515	0.3659	0.062*	
C5	0.3508 (12)	0.3775 (9)	0.4323 (6)	0.040 (2)	
C6	0.3390 (15)	0.2991 (10)	0.4931 (8)	0.054 (3)	
H6	0.3745	0.2151	0.4925	0.065*	
C7	0.2778 (17)	0.3444 (11)	0.5512 (8)	0.059 (3)	
H7	0.2710	0.2909	0.5901	0.071*	
C8	0.2221 (14)	0.4732 (10)	0.5553 (6)	0.045 (2)	
C9	0.1555 (16)	0.5221 (12)	0.6139 (6)	0.052 (3)	
H9	0.1428	0.4704	0.6531	0.063*	
C10	0.1082 (17)	0.6457 (15)	0.6147 (7)	0.060 (3)	
H10	0.0646	0.6789	0.6546	0.072*	
C11	0.1255 (15)	0.7242 (11)	0.5544 (6)	0.049 (2)	
C12	0.078 (2)	0.8630 (13)	0.5530 (8)	0.068 (4)	
H12A	−0.0089	0.8790	0.5129	0.081*	
H12B	0.1773	0.9133	0.5483	0.081*	
H12C	0.0340	0.8847	0.5970	0.081*	
C13	0.2361 (12)	0.5538 (9)	0.4964 (5)	0.0353 (18)	
C14	0.2975 (11)	0.5055 (9)	0.4335 (6)	0.038 (2)	

### Atomic displacement parameters (Å^2^)

**Table:**

	*U*^11^	*U*^22^	*U*^33^	*U*^12^	*U*^13^	*U*^23^
Hg1	0.0797 (3)	0.0314 (2)	0.0497 (3)	0.00853 (18)	0.0099 (2)	0.00815 (17)
Br1	0.0775 (9)	0.1107 (13)	0.0618 (9)	0.0161 (9)	0.0063 (7)	0.0348 (9)
Br2	0.1172 (12)	0.0377 (6)	0.0795 (10)	−0.0148 (7)	0.0114 (9)	0.0107 (6)
N1	0.059 (5)	0.029 (4)	0.039 (4)	−0.004 (3)	0.002 (4)	−0.004 (3)
N2	0.046 (4)	0.034 (4)	0.033 (4)	0.002 (3)	0.005 (3)	0.001 (3)
C1	0.148 (16)	0.056 (8)	0.059 (9)	0.013 (9)	0.030 (10)	−0.006 (7)
C2	0.062 (6)	0.038 (5)	0.029 (5)	0.000 (4)	0.003 (4)	−0.005 (4)
C3	0.080 (8)	0.040 (6)	0.050 (6)	0.001 (5)	0.016 (6)	−0.015 (5)
C4	0.046 (6)	0.034 (5)	0.074 (8)	0.005 (4)	0.004 (5)	−0.007 (5)
C5	0.037 (5)	0.031 (4)	0.050 (6)	0.002 (4)	−0.005 (4)	0.001 (4)
C6	0.055 (6)	0.034 (5)	0.071 (8)	0.006 (4)	−0.001 (6)	0.015 (5)
C7	0.073 (8)	0.036 (6)	0.064 (8)	−0.006 (5)	−0.002 (6)	0.023 (5)
C8	0.050 (6)	0.041 (5)	0.041 (5)	−0.010 (4)	−0.002 (4)	0.013 (4)
C9	0.064 (7)	0.052 (6)	0.039 (6)	−0.008 (5)	0.000 (5)	0.007 (5)
C10	0.059 (7)	0.078 (9)	0.045 (6)	−0.015 (6)	0.020 (5)	−0.013 (6)
C11	0.057 (6)	0.045 (6)	0.043 (6)	0.001 (5)	0.001 (5)	−0.002 (4)
C12	0.102 (11)	0.048 (7)	0.055 (7)	0.008 (7)	0.014 (7)	−0.008 (6)
C13	0.036 (4)	0.032 (4)	0.037 (5)	−0.003 (3)	−0.001 (4)	0.006 (4)
C14	0.031 (4)	0.035 (4)	0.045 (5)	−0.001 (3)	−0.005 (4)	0.005 (4)

### Geometric parameters (Å, °)

**Table:**

Hg1—Br2	2.5053 (16)	C6—H6	0.9300
Hg1—Br1	2.5156 (17)	C7—C8	1.433 (16)
N1—Hg1	2.345 (8)	C7—H7	0.9300
N2—Hg1	2.340 (8)	C8—C9	1.377 (17)
C1—C2	1.474 (19)	C8—C13	1.409 (13)
C1—H1A	0.9600	C9—C10	1.36 (2)
C1—H1B	0.9600	C9—H9	0.9300
C1—H1C	0.9600	C10—C11	1.422 (18)
C2—N1	1.359 (13)	C10—H10	0.9300
C2—C3	1.403 (15)	C11—N2	1.330 (14)
C3—C4	1.370 (18)	C11—C12	1.512 (18)
C3—H3	0.9300	C12—H12A	0.9600
C4—C5	1.400 (17)	C12—H12B	0.9600
C4—H4	0.9300	C12—H12C	0.9600
C5—C14	1.416 (13)	C13—N2	1.365 (12)
C5—C6	1.421 (16)	C13—C14	1.425 (15)
C6—C7	1.33 (2)	C14—N1	1.333 (13)
			
Br2—Hg1—Br1	116.23 (6)	C7—C6—C5	120.9 (10)
N1—Hg1—Br1	109.6 (2)	C7—C6—H6	119.6
N1—Hg1—Br2	115.7 (2)	C5—C6—H6	119.6
N2—Hg1—Br1	117.3 (2)	C6—C7—C8	122.0 (11)
N2—Hg1—Br2	118.2 (2)	C6—C7—H7	119.0
N2—Hg1—N1	71.2 (3)	C8—C7—H7	119.0
C14—N1—C2	121.8 (9)	C9—C8—C13	118.3 (10)
C14—N1—Hg1	115.7 (7)	C9—C8—C7	123.4 (11)
C2—N1—Hg1	122.5 (7)	C13—C8—C7	118.4 (11)
C11—N2—C13	119.4 (9)	C10—C9—C8	120.2 (11)
C11—N2—Hg1	125.3 (7)	C10—C9—H9	119.9
C13—N2—Hg1	115.2 (6)	C8—C9—H9	119.9
C2—C1—H1A	109.5	C9—C10—C11	119.8 (12)
C2—C1—H1B	109.5	C9—C10—H10	120.1
H1A—C1—H1B	109.5	C11—C10—H10	120.1
C2—C1—H1C	109.5	N2—C11—C10	120.8 (11)
H1A—C1—H1C	109.5	N2—C11—C12	117.3 (11)
H1B—C1—H1C	109.5	C10—C11—C12	121.9 (11)
N1—C2—C3	119.4 (10)	C11—C12—H12A	109.5
N1—C2—C1	119.9 (10)	C11—C12—H12B	109.5
C3—C2—C1	120.7 (11)	H12A—C12—H12B	109.5
C4—C3—C2	119.3 (11)	C11—C12—H12C	109.5
C4—C3—H3	120.3	H12A—C12—H12C	109.5
C2—C3—H3	120.3	H12B—C12—H12C	109.5
C3—C4—C5	121.5 (10)	N2—C13—C8	121.5 (10)
C3—C4—H4	119.3	N2—C13—C14	118.4 (8)
C5—C4—H4	119.3	C8—C13—C14	120.1 (9)
C4—C5—C14	116.7 (10)	N1—C14—C5	121.3 (10)
C4—C5—C6	123.8 (10)	N1—C14—C13	119.4 (9)
C14—C5—C6	119.5 (10)	C5—C14—C13	119.2 (9)
			
N1—C2—C3—C4	1.0 (18)	C13—C14—N1—C2	−179.8 (9)
C1—C2—C3—C4	178.4 (13)	C5—C14—N1—Hg1	179.7 (7)
C2—C3—C4—C5	−0.8 (19)	C13—C14—N1—Hg1	−2.0 (11)
C3—C4—C5—C14	1.0 (16)	C3—C2—N1—C14	−1.6 (16)
C3—C4—C5—C6	179.8 (11)	C1—C2—N1—C14	−178.9 (12)
C4—C5—C6—C7	−178.5 (11)	C3—C2—N1—Hg1	−179.2 (9)
C14—C5—C6—C7	0.3 (17)	C1—C2—N1—Hg1	3.4 (16)
C5—C6—C7—C8	−0.3 (19)	C10—C11—N2—C13	−0.4 (16)
C6—C7—C8—C9	178.8 (12)	C12—C11—N2—C13	178.6 (10)
C6—C7—C8—C13	−1.1 (18)	C10—C11—N2—Hg1	176.5 (8)
C13—C8—C9—C10	−1.3 (17)	C12—C11—N2—Hg1	−4.5 (15)
C7—C8—C9—C10	178.8 (12)	C8—C13—N2—C11	0.0 (15)
C8—C9—C10—C11	0.9 (19)	C14—C13—N2—C11	178.2 (9)
C9—C10—C11—N2	0.0 (19)	C8—C13—N2—Hg1	−177.2 (7)
C9—C10—C11—C12	−178.9 (12)	C14—C13—N2—Hg1	1.0 (11)
C9—C8—C13—N2	0.9 (15)	C11—N2—Hg1—N1	−178.4 (9)
C7—C8—C13—N2	−179.2 (10)	C13—N2—Hg1—N1	−1.4 (6)
C9—C8—C13—C14	−177.3 (9)	C11—N2—Hg1—Br2	71.9 (9)
C7—C8—C13—C14	2.6 (15)	C13—N2—Hg1—Br2	−111.1 (6)
C4—C5—C14—N1	−1.6 (14)	C11—N2—Hg1—Br1	−75.5 (9)
C6—C5—C14—N1	179.6 (9)	C13—N2—Hg1—Br1	101.6 (6)
C4—C5—C14—C13	−179.9 (9)	C14—N1—Hg1—N2	1.7 (7)
C6—C5—C14—C13	1.2 (14)	C2—N1—Hg1—N2	179.6 (9)
N2—C13—C14—N1	0.7 (14)	C14—N1—Hg1—Br2	114.7 (7)
C8—C13—C14—N1	178.9 (9)	C2—N1—Hg1—Br2	−67.5 (8)
N2—C13—C14—C5	179.1 (8)	C14—N1—Hg1—Br1	−111.4 (7)
C8—C13—C14—C5	−2.7 (14)	C2—N1—Hg1—Br1	66.4 (8)
C5—C14—N1—C2	1.9 (15)		

### Hydrogen-bond geometry (Å, °)

**Table:**

*D*—H···*A*	*D*—H	H···*A*	*D*···*A*	*D*—H···*A*
C1—H1C···Br2^i^	0.96	2.85	3.812 (18)	178

Symmetry codes: (i) −*x*+1, *y*−1/2, −*z*+1/2.
